# Potentiation of B2 receptor signaling by AltB2R, a newly identified alternative protein encoded in the human bradykinin B2 receptor gene

**DOI:** 10.1016/j.jbc.2021.100329

**Published:** 2021-01-23

**Authors:** Maxime Gagnon, Martin Savard, Jean-François Jacques, Ghassan Bkaily, Sameh Geha, Xavier Roucou, Fernand Gobeil

**Affiliations:** 1 Department of Biochemistry, Université de Sherbrooke, Sherbrooke, Québec, Canada; 2 Institute of Pharmacology, Université de Sherbrooke, Sherbrooke, Québec, Canada; 3 Department of Pharmacology & Physiology, Université de Sherbrooke, Sherbrooke, Québec, Canada; 4 Department of Immunology & Cellular Biology, Université de Sherbrooke, Sherbrooke, Québec, Canada; 5 Department of Pathology, Centre Hospitalier Universitaire de Sherbrooke, Sherbrooke, Québec, Canada

**Keywords:** alternative open reading frames (AltORFs), coding DNA sequence (CDS), G-protein-coupled receptors (GPCRs), bradykinin (BK), B2 receptor (B2R), signal transduction, mitogen-activated protein kinases (MAPK), AltORFs, alternative open reading frames, BiFC, bimolecular fluorescence complementation, BK, bradykinin, CDC, coding DNA sequence, DAB, diaminobenzidine, DMEM, Dulbecco's modified Eagle's medium, EGFR, epidermal growth factor receptor, FBS, fetal bovine serum, GPCR, G-protein-coupled receptor, IF, immunofluorescence, IHC, immunohistochemistry, IP_3_, 1,4,5-inositol trisphosphate, KD, kallidin, MAPK, mitogen-activated protein kinases, PBS, phosphate-buffered saline, WB, western blot

## Abstract

Recent functional and proteomic studies in eukaryotes (www.openprot.org) predict the translation of alternative open reading frames (AltORFs) in mature G-protein-coupled receptor (GPCR) mRNAs, including that of bradykinin B2 receptor (B2R). Our main objective was to determine the implication of a newly discovered AltORF resulting protein, termed AltB2R, in the known signaling properties of B2R using complementary methodological approaches. When ectopically expressed in HeLa cells, AltB2R presented predominant punctate cytoplasmic/perinuclear distribution and apparent cointeraction with B2R at plasma and endosomal/vesicular membranes. The presence of AltB2R increases intracellular [Ca^2+^] and ERK1/2-MAPK activation (*via* phosphorylation) following B2R stimulation. Moreover, HEK293A cells expressing mutant B2R lacking concomitant expression of AltB2R displayed significantly decreased maximal responses in agonist-stimulated Gα_q_-Gα_i2/3_–protein coupling, IP_3_ generation, and ERK1/2-MAPK activation as compared with wild-type controls. Conversely, there was no difference in cell-surface density as well as ligand-binding properties of B2R and in efficiencies of cognate agonists at promoting B2R internalization and β-arrestin 2 recruitment. Importantly, both AltB2R and B2R proteins were overexpressed in prostate and breast cancers, compared with their normal counterparts suggesting new associative roles of AltB2R in these diseases. Our study shows that *BDKRB2* is a dual-coding gene and identifies AltB2R as a novel positive modulator of some B2R signaling pathways. More broadly, it also supports a new, unexpected alternative proteome for GPCRs, which opens new frontiers in fields of GPCR biology, diseases, and drug discovery.

Until recently, it was believed that overlapping open reading frames (ORFs) and polycistronic mRNAs only occurred in prokaryotes and viruses. However, recent studies have challenged the notion of an mRNA coding for a single annotated protein and showed the pervasive properties of the translation machinery (1). Multiple examples of mRNAs coding for an annotated coding sequence (RefORF) and an alternative open reading frame (AltORF) have been published in recent years (2, 3, 4, 5, 6, 7, 8, 9, 10, 11, 12, 13, 14, 15), such as uMKKS1-2 (9), AltMiD51 (6), AltPrP (3), AltATXN1 (4), AltHNRNPUL1 (8), ARF (10), ALEX (11, 15), MUC1-ARF (12), AltRPP14 (13). About 183,000 AltORFs are predicted in the human transcriptome, of which 76% are predicted to be encoded in coding RNAs (6), the remainder being predicted to be encoded on RNAs previously annotated as noncoding. These AltORFs localize in the 5'/3'-UTRs or overlap the annotated coding sequence (CDS) in the +2 or the +3 reading frames. Several specific examples have been reported in recent years, the first one being ALEX, an AltORF encoded in the coding sequence of the G protein XLαs (11, 15). ALEX interacts with XLαs and prevents it from activating the adenylate cyclase. More recently, the rat *Adora2a* gene has been shown to code for the adenosine A_2a_ receptor and a novel alternative protein encoded in an AltORF located in the 5’UTR, named uORF5. Expression of uORF5 modulates the expression of several genes involved in the mitogen-activated protein kinases (MAPK)/ERK pathway, and stimulation of the A_2a_R receptor results in an increase in uORF5 expression (7). Another recent example is the discovery of the seven amino acid peptide named PEP7, which is encoded in an AltORF present in the 5'UTR of the rat angiotensin AT_1a_R gene (14). Exogenously applied PEP7 inhibited β-arrestin-dependent MAPK activation in HEK293 cells expressing AT_1a_R. *In vivo*, PEP7 blocked angiotensin II-stimulated saline intake (14).

Using the Openprot database (16), we identified a total of 4645 putative novel AltORFs with a minimum length of 30 codons in 81% of the 830 human G-protein-coupled receptor (GPCR) genes (Fig. 1*A*; see Table S1 for complete list of AltORFs). In total, 42% of them are localized in the 3'UTR, 23% in the 5'UTR, and more than 35% are predicted to overlap the annotated GPCR coding sequence.

**Figure 1:**
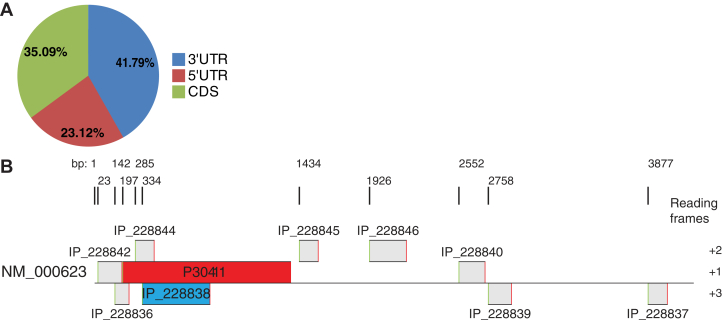
**Predicted AltORFs in the human GPCRome.***A*, pie chart showing the distribution of localization of the predicted AltORFs in human GPCR genes. AltORFs^3'UTR^ are in *blue*, AltORFs^5'UTR^ are in *red*, AltORFs^CDS^ are in *green*. Graph generated from 2019-01-09 AltORFs prediction.xlsx (first tab); search result generated on Openprot.org (release 1.3) by searching with the gene name of every human GPCR annotated on Uniprot.org as of January 9th, 2019. *B*, representation of the mRNA of the *BDKRB2* gene and its predicted AltORFs to scale. The coding sequence for B2R is in *red*, the coding sequence for AltB2R is in *blue* and other AltORFs are in *gray* (second tab).

The human *BDKRB2* gene has nine predicted AltORFs (Fig. 1*B*, Table S2). One of them is a 474 nucleotide AltORF overlapping the coding sequence of B2R in the +3 reading frame (Fig. 1*B*). This AltORF, which we termed AltB2R, is predicted to produce a 157 amino acid long protein with a 17 kDa molecular weight and an isoelectric point of 6.58 (ProtParam tool, ExPASy server). PSORT bioinformatic analysis indicates that AltB2R is likely to be a soluble cytosolic protein rather than a transmembrane protein (not shown).

B2R exhibits constitutive expression in various tissues/organs and exerts pleiotropic (beneficial and deleterious) effects in various physiopathological conditions, such as diabetes, cardiovascular diseases, and cancers (17, 18, 19, 20, 21). Its principal peptide agonist ligands are the nona- and decapeptide bradykinin and Lys-BK (alias kallidin; KD), respectively. Whereas the carboxy-terminally truncated peptide metabolites desArg^9^-BK and desArg^10^-KD, derived from enzymatic activities of carboxypeptidases M/N, selectively bind to the other type of kinin receptor, the inducible B1R (22). B2R can initiate multiple signaling pathways through multiple G proteins (*e.g.*, Gα_q/11_, Gα_12/13_, Gα_i_ and Gα_s_) and β-Arrestins (βarrs) in mammalian cells (22, 23, 24). These latter are often linked to successive activation of MAPK/ERK1/2 involved in transcription of genes that promote cellular proliferation and survival (22, 25, 26).

In this study, we investigated whether the expression of AltB2R can directly regulate B2R activity. Our results demonstrated that AltB2R can interact with B2R protein and enhance B2R-mediated transduction signals occurring through Gα_q/i2/i3_ signaling activity. We also showed *bona fide* endogenous coexpression of AltB2R and B2R with variable expression patterns, in clinical specimens of different types of solid human cancers, indicating that it is likely to perform important functions in these diseases.

## Results

### Coexpression and colocalization of B2R and AltB2R

**Figure 2:**
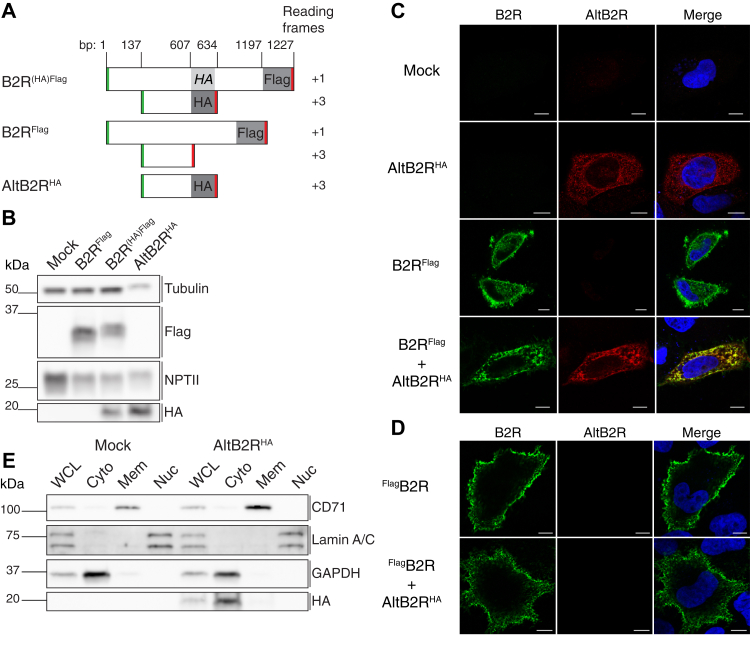
**AltB2R and B2R are coexpressed from the same coding sequence and colocalize in the cytoplasm and membranes of HeLa cells.***A*, strategy used to detect AltB2R and B2R. B2R with an in-frame C-terminal Flag tag, without and with an out-of-frame C-terminal HA tag on AltB2R within the coding sequence of B2R, and AltB2R with an in-frame C-terminal tag without the reference B2R. Positions of start and stop codons are indicated in *green* and *red*, respectively. Construction drawings are not to scale. *B*, detection of B2R^Flag^ and AltB2R^HA^ by western blotting using anti-Flag and anti-HA antibodies. Tubulin serves as a loading control and NPTII serves as a transfection control. Representative western blot of two independent experiments performed. *C*, subcellular localization of both B2R^Flag^ and AltB2R^HA^ by immunofluorescence using primary antibodies as in *B*. B2R^Flag^ (*green*) and AltB2R^HA^ (*red*) staining is shown in punctate granular vesicle-like structures within cytoplasm, perinuclear area, and underneath the plasma membrane. Scale bar: 10 μm. *D*, expression of B2R with an N-terminal Flag tag (^Flag^B2R) in live, nonpermeabilized cells confirmed localization at the plasma membrane. As expected, no immunofluorescent labeling of intracellular AltB2R^HA^ was observed in nonpermeabilized conditions. Scale bar: 10 μm. *C* and *D*, representative single confocal images (midsection of cells) from three independent experiments. *E*, subcellular fractionation shows an enrichment of AltB2R^HA^ in the cytoplasmic fraction compared with the whole cell lysat (WCL). Immunoblots of CD71 (plasma membrane; Mem), Lamin A/C (nucleus; Nuc), and GAPDH (cytoplasm; Cyto) were used to assess the purity of each fraction. Representative western blot of two independent experiments performed.

To test whether AltB2R is coexpressed with B2R, an HA tag was introduced in frame with AltB2R to produce carboxy-tagged AltB2R (AltB2R^HA^), within the B2R coding sequence. Since B2R has a C-terminal Flag tag, this construct is termed B2R^(HA)Flag^ to indicate that the HA tag is silent within the B2R reading frame (Fig. 2*A*). Control constructs included B2R^Flag^ and AltB2R^HA^. B2R^Flag^ and AltB2R^HA^ were detected in cells transfected with B2R^(HA)Flag^, indicating that both proteins are expressed from the coding sequence of B2R (Fig. 2*B*). Immunofluorescence staining in permeabilized HeLa cells transfected with AltB2R^HA^ showed the cytoplasmic localization of the protein with a nonhomogeneous distribution (Fig. 2*C*). Cotransfection with B2R^Flag^ indicated that both proteins colocalized at the cell membrane, perinuclear regions, and in cytoplasmic foci, most likely to be endosome and/or plasma membrane transport vesicles (Fig. 2*C*). Similar experiments conducted in nonpermeabilized cell conditions gave strong punctate surface staining of the B2R (Fig. 2*D*). Further confirmation of the cytoplasmic localization of AltB2R was obtained by western blot analysis of highly purified subcellular fractions (nuclei, cytoplasm, and cell membrane fractions) (Fig. 2*E*).

### B2R/AltB2R interaction in living cells

**Figure 3:**
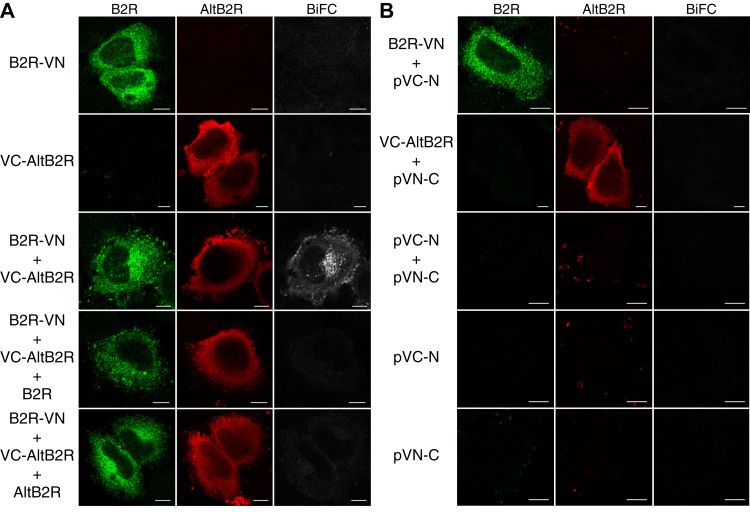
**AltB2R and B2R interaction shown by bimolecular fluorescence complementation (BiFC) in HeLa cells.***A*, expression of B2R^Flag^-VN and VC-AltB2R^HA^ results in BiFC signals in transfected HeLa cells. BiFC signals is lost when competing B2R^Flag^ or AltB2R^HA^ interacts with available pool of VC-AltB2R^HA^ or B2R^Flag^-VN, respectively. *B*, no detectable BiFC signals from nonspecific assembly of the Venus protein. Scale bar: 10 μm. *A* and *B*, representative single confocal mid-section images from three independent experiments are shown.

Cytoplasmic colocalization of B2R and AltB2R suggested a possible interaction between both proteins. To test this hypothesis, we used bimolecular fluorescence complementation (BiFC) based on the association of split VENUS fragments composed of VN (amino acids 1–210) and VC (amino acids 210–238). B2R^Flag^-VN and VC-AltB2R^HA^ displayed a membranous/cytoplasmic localization (Fig. 3*A*), in accordance with our previous microscopy and western blot data. Cells cotransfected with B2R^Flag^-VN and VC-AltB2R^HA^ displayed Venus fluorescence, confirming B2R/AltB2R interaction *in cellulo* (Fig. 3*A* and Fig. S3*B*). In support of a specific interaction between B2R and AltB2R, competition with B2R lacking the VN fragment or AltB2R lacking the VC fragment completely prevented the formation of BiFC complexes (Fig. 3*A*). Further experiments performed with constructs where the Venus fragments were switched around to B2R^Flag^-VC and VN-AltB2R^HA^ gave similar positive results (data not shown). In control experiments, cells expressing either VN or VC, VN and VC, B2R-VN and VC, or VC-AltB2R and VN did not display any BiFC signal (Fig. 3*B*). To demonstrate the specificity of interaction between AltB2R and B2R, we conducted similar experiments in cells transfected with the human B1R^Flag^-VC and VN-AltB2R^HA^. In this experimental setting, no BiFC signal could be detected between the two proteins, suggesting some degree of specificity in the association between AltB2R and B2R (Fig. S3*A*).

### AltB2R enhances MAPK ERK1/2 activation and calcium mobilization following B2R stimulation

**Figure 4:**
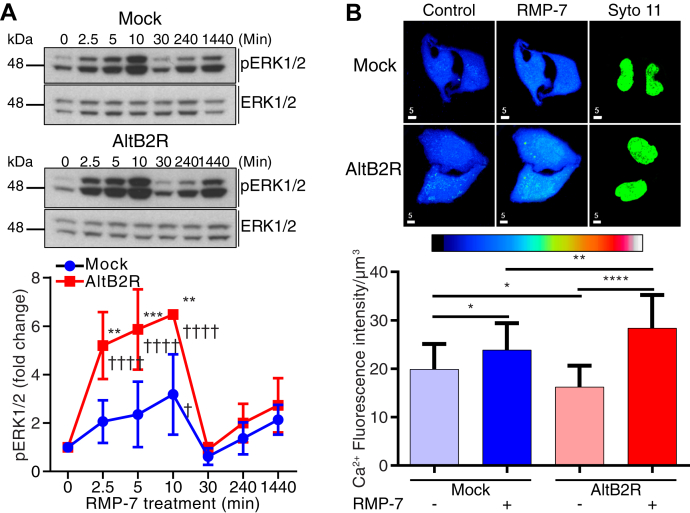
**Stable AltB2R**^**Flag**^**expressing HeLa cells show increased calcium release and MAPK activation compared with stable Mock cells in response to B2R stimulation.***A*, representative western blot images showing the time course of ERK1/2 phosphorylation after stimulation with 1 μM RMP-7 in Mock *versus* AltB2R transfected cells (*top*). Quantitative densitometry analysis of pERK1/2 levels over time (*bottom*). P-ERK1/2 levels were normalized to total ERK at each time point and were expressed as fold changes over basal levels (in unstimulated cells). Data represent mean ± SD, n = 3, multiple comparison *versus* corresponding time point using two-way ANOVA with Sidak’s correction, ∗∗*p* < 0.01, ∗∗∗*p* < 0.001, multiple comparison *versus* control (0 min) using two-way ANOVA with Dunnett's correction, ^†^*p* < 0.05, ^††††^*p* < 0.0001. *B*, representative confocal images of B2R-mediated intracellular calcium mobilization between Mock and AltB2R^Flag^ stable cell lines before and after 1 μΜ RMP-7 stimulation (*top*). Pseudo color bar shows fluorescence intensity scale of calcium ranging from 0 (*black*) to 255 (*white*) RGB values. Nuclear identity and delineation were established at the end of experiments with the nucleic acid fluorescent dye Syto-11. Scale bar: 5 μm. Quantification of calcium mobilization in AltB2R^Flag^ stable cells compared with Mock (*bottom*). Data represent mean ± SD, n = 3, multiple comparison using one-way ANOVA with Sidak’s correction, ∗*p* < 0.05, ∗∗*p* < 0.01, ∗∗∗∗*p* < 0.0001.

Next, we tested if the physical interaction between AltB2R and B2R could modulate B2R signaling processes. Considering that many signaling pathways of B2R converge toward the activation of MAPK ERK1/2 (27), we first studied the level of phosphorylation of ERK1/2 upon agonist stimulation of endogenous B2R in HeLa cells. The metabolically stabilized, selective, potent B2R agonists NG291 and RMP-7 were used in these functional assays and in others to avoid possible interference and unpredictable actions of peptidases on B2R signaling (ex. formation of active metabolites at receptors other than B2R, cross talk between peptidases and B2R) in the cell models employed for the study, *i.e.*, HeLa and HEK293 cells (28, 29). In mock-HeLa cells stimulated with RMP-7, levels of phospho-ERK1/2 reached a maximum 10 min poststimulation and returned to basal levels after 30 min (Fig. 4*A*). ERK1/2 phosphorylation kinetics were similar in HeLa cells stably expressing AltB2R, but maximum levels at 10 min were significantly higher compared with mock cells (Fig. 4*A*). We performed the same assays using instead the biostable, selective B1R agonist NG29 (30) and did not observe any difference in the increased levels of phosphorylated ERK1/2 between mock and AltB2R^Flag^ cells (Fig. S3), indicating some functional specificity of AltB2R toward the modulation of MAPK from B2R. Since B2R-induced MAPK ERK1/2 activation often requires the mobilization of intracellular calcium (i[Ca^2+^]), we then analyzed whether the presence of AltB2R can also impact intensity of intracellular Ca^2+^ mobilization in B2R-stimulated cells previously loaded with the fluorescent Ca^2+^-sensitive dye Fluo-4. In mock stable cells, RMP-7 treatment induced a significant increase in i[Ca^2+^] (Fig. 4*B*). In AltB2R^Flag^-expressing cells, basal levels of free Ca^2+^ were significantly lower compared with mock cells. Interestingly, these latter cells had significantly higher Ca^2+^ mobilization levels compared with mock cells in response to RMP-7 (Fig. 4*B*). From these observations, it could be inferred that the potentiation of B2R agonist responses elicited by AltB2R may involve upstream targets of MAPK/ERK and Ca^2+^ pathways.

### The lack of AltB2R expression reduces MAPK activation and IP_3_ generation following B2R stimulation

In order to further substantiate the magnitude of effects of AltB2R on the pharmacological and signaling properties of B2R, we generated a mutant construct termed B2R^(Ø)^, expressed in HEK293A cells (Fig. S1*A*). This construct is a monocistronic version of B2R with the mutation C168T. In this mutant, a premature stop codon is introduced in the AltB2R coding sequence (Q11stop) in a manner synonymous for B2R (L56 L). Thus, the B2R encodes B2R and AltB2R, whereas mutant B2R^(Ø)^ encodes only B2R. Indeed, western blotting showed that cells transfected with B2R^(Ø)^ did not express AltB2R, while having relatively unaltered B2R expression compared with mutant cells encoding both B2R/AtlB2R (Fig. S1*B*).

**Figure 5:**
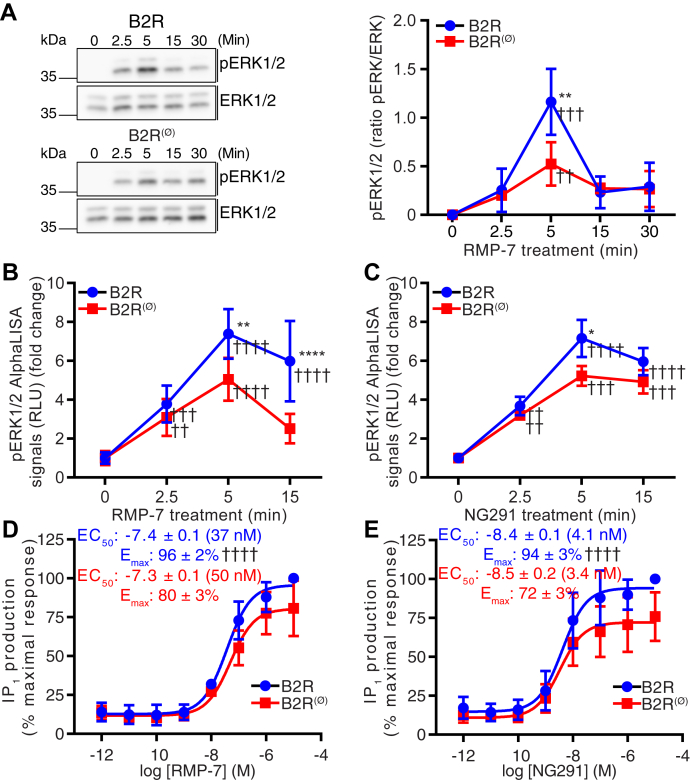
**HEK293A cells transiently transfected with B2R**^**(Ø)**^**mutant (lacking AltB2R) show decreased maximal IP**_**1**_**generation and MAPK activation compared with B2R transfected cells following agonist stimulation.***A*, representative western blot images showing the time course of ERK1/2 phosphorylation after stimulation with 1 μM RMP-7 in HEK293A cells transfected with B2R and B2R^(Ø)^ (*top left*). Quantitative densitometry analysis of pERK1/2 levels over time (*top right*). pERK/total ERK ratios were calculated for each time point. Data represent mean ± SD, n = 3, multiple comparison *versus* corresponding time point using two-way ANOVA with Sidak's correction, ∗∗*p* < 0.01, multiple comparison *versus* control (0 min) using two-way ANOVA with Dunnett's correction, ^††^*p* < 0.01, ^†††^*p* < 0.001. *B* and *C*, time-course of pERK1/2 AlphaLISA signals after B2R stimulation with 1 μM RMP-7 (*B*) or NG291 (*C*) in B2R and B2R^(Ø)^-transfected HEK293A cells. No significant differences were observed in basal pERK1/2 levels (0 min time point) between unstimulated B2R-HEK293A and B2R^(Ø)^-HEK293A cells (not shown). Data represent mean ± SD, n = 2, multiple comparison *versus* corresponding time point using two-way ANOVA with Sidak’s correction, ∗*p* < 0.05, ∗∗*p* < 0.01, multiple comparison *versus* control (0 min) using two-way ANOVA with Dunnett’s correction, ^††^*p* < 0.01, ^†††^*p* < 0.001, ^††††^*p* < 0.0001. *D* and *E*, IP_1_ production after RMP-7 (*D*) or NG291 (*E*) stimulation in HEK293A cells transfected with B2R and B2R^(Ø)^. Data represent mean ± SD, n = 5, 4; unpaired *t*-test for EC_50_ and E_max_, ^††††^*p* < 0.0001.

Western blotting experiments showed that in B2R-expressing cells stimulated with RMP-7, levels of phospho-ERK1/2 reached a maximum at 5 min poststimulation and returned to basal levels after 15 min (Fig. 5*A*). ERK1/2 phosphorylation kinetics were similar in cells transfected with B2R^(Ø)^, but maximal levels of phospho-ERK1/2 were significantly lower (Fig. 5*A*). To consolidate the western blot data, AlphaLISA assays were also conducted in parallel in the same cell lines for the quantitative analyses of B2R-stimulated ERK1/2 phosphorylation (Fig. 5, *B* and *C*). Again, the results showed decreased maximal levels of phospho-ERK1/2 in B2R^(Ø)^ *versus* B2R-transfected cells when stimulated with either RMP-7 or NG291. Since calcium mobilization precedes ERK1/2 phosphorylation and results from 1,4,5-inositol trisphosphate (IP_3_) binding to IP_3_R at the endoplasmic reticulum membrane, we tested whether the presence AltB2R could also amplify production of IP3 resulting from B2R activation. IP_3_ formation (indirectly measured *via* its stabilized metabolite IP_1_) was assessed following generation of full concentration-response profiles of B2R agonists RMP-7 and NG291 in B2R and B2R^(Ø)^-transfected cells. Results indicated no significant difference in potencies (EC_50_ values) of agonists between B2R- and B2R^(Ø)^-transfected cells (Fig. 5, *D* and *E*). However, significantly lower agonist efficacies (Emax values) were noted in cells without AltB2R expression (16 and 22% reduction of efficacy for RMP-7 and NG291, respectively). These results indicate that AltB2R may impact B2R signal transduction pathways at various levels.

### AltB2R modulates G protein subtype coupling efficacy to but not internalization rate of B2R

**Figure 6:**
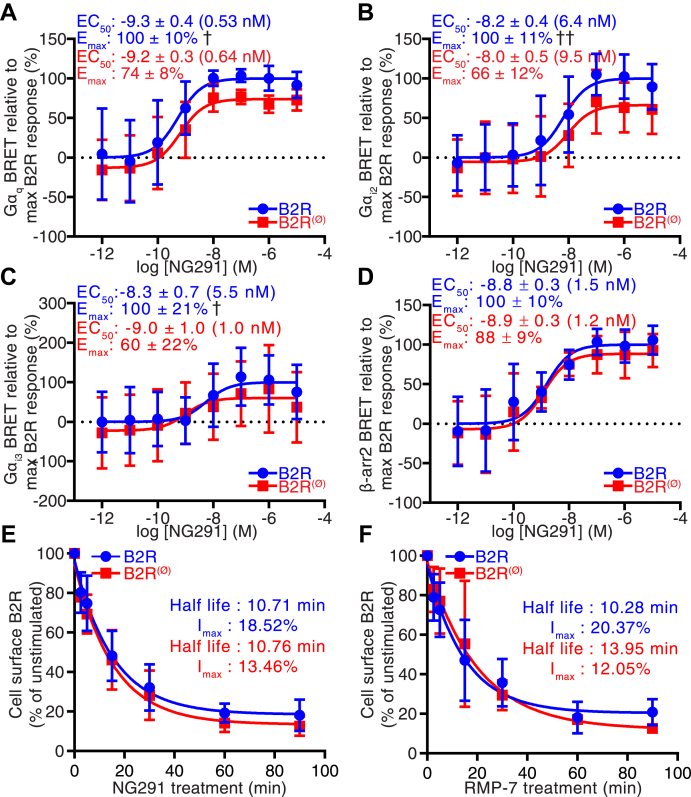
**HEK293A cells transiently transfected with B2R**^**(Ø)**^**mutant (lacking AltB2R) show no difference in receptor internalization kinetics but show decreased G protein coupling efficiencies following agonist stimulation.***A*–*C*, G protein coupling efficiency after stimulation with NG291 in HEK293A cells transfected with B2R and B2R^(Ø)^, by following BRET proximity of Gα_q_-RLuc2 and GFP10-Gγ_1_ (*A*), Gα_i2_-RLuc2 and GFP10-Gγ_1_ (*B*), or Gα_i3_-RLuc2 and GFP10-Gγ_1_ (*C*). Data represent mean ± SD, n = 3, 4, 4, unpaired *t*-test for EC_50_ (not significant) and E_max_, ^†^*p* < 0.05, ^††^*p* < 0.01. *D*, β-arrestin2 recruitment at the plasma membrane after stimulation with NG291 in HEK293A cells transfected with B2R and B2R^(Ø)^ by following BRET proximity of β-arr2-RLuc2 and rGFP-CAAX. Data represent mean ± SD, n = 3, unpaired t-test for EC_50_ and E_max,_ not significant. *E* and *F*, internalization kinetics of B2R by cell surface ELISA after stimulation with 1 μM RMP-7 (*E*) or NG291 (*F*) in HEK293A cells transfected with ^Flag^B2R or ^Flag^B2R^(Ø)^, respectively. Note. B2R-HEK293A and B2R(Ø)-HEK293A cells gave equivalent signals at time zero (unstimulated) (not shown). Data represent mean ± SD, n = 3, 3, multiple comparison *versus* corresponding time point using two-way ANOVA with Sidak's correction, unpaired *t*-test for I_max_ (maximal internalization) and half-life, not significant.

The observation that AltB2R is necessary to achieve a full production of IP_3_ upon B2R stimulation suggests that AltB2R modulates coupling between B2R and Gα_q_ and/or Gα_i_. To test this hypothesis, we used live-cell bioluminescence resonance energy transfer (BRET) measurements to monitor the activation of G proteins by B2R. HEK293A cells were cotransfected with vectors coding for Gα_q_-RlucII or Gα_i2/3_-RlucII, Gβ, GFP10-Gγ_1_, and B2R or B2R^(Ø)^. In this experimental setting, the dissociation of Gα_q_-RlucII or Gα_i2/3_-RlucII from GFP10-Gγ_1_ results in a decrease of BRET signals. BRET data in Figure 6, *A*–*C* are presented relative to the maximum B2R-induced BRET response. We observed no difference of the EC_50_ for the dissociation Gα_q_/Gγ_1_ in B2R-transfected cells compared with B2R^(Ø)^-transfected cells (Fig. 6*A*). There was a significant decrease in the maximal dissociation E_max_ (100 ± 10% *versus* 74 ± 8% for B2R and B2R^(Ø)^, respectively). For the dissociation Gα_i2_/Gγ_1_, we observed no difference in the EC_50_ whether AltB2R was present or absent, but E_max_ decreased from 100 ± 11% in B2R-transfected cells to 66 ± 12% in B2R^(Ø)^-transfected cells (Fig. 6*B*). For the dissociation of Gα_i3_/Gγ_1_, the EC_50_ was similar in B2R- and B2R^(Ø)^-transfected cells, but E_max_ was significantly lower in B2R^(Ø)^-transfected cells (60 ± 22% *versus* 100 ± 21%) (Fig. 6*C*).

Since both G proteins and β-arrestins commonly participate in activation of MAPK pathways, we extended the BRET analysis to determine the effects of AltB2R on the efficacy of β-arrestin2 recruitment at the plasma membrane of HEK293A cells transfected with vectors coding for β-arrestin2-RLucII, rGFP-CAAX, and B2R or B2R^(Ø)^. BRET signals were monitored after addition of NG291. We detected no difference in the recruitment of β-arrestin2 in cells either expressing or not AltB2R (Fig. 6*D*); similar results were obtained with BK (data not shown). Moreover, AltB2R did not impair both the magnitude and rate of agonist-promoted internalization of B2R, most likely dependent on β-arrestin2 (31) (Fig. 6, *E* and *F*). Hence, these results suggest that the AltB2R activity-dependent changes in MAPK activation rely primarily on a select subsets of G proteins (*i.e.*, Gα_q_- Gα_i2/3_-proteins).

### AltB2R does not affect cell-surface density and ligand-binding properties of B2R

**Figure 7:**
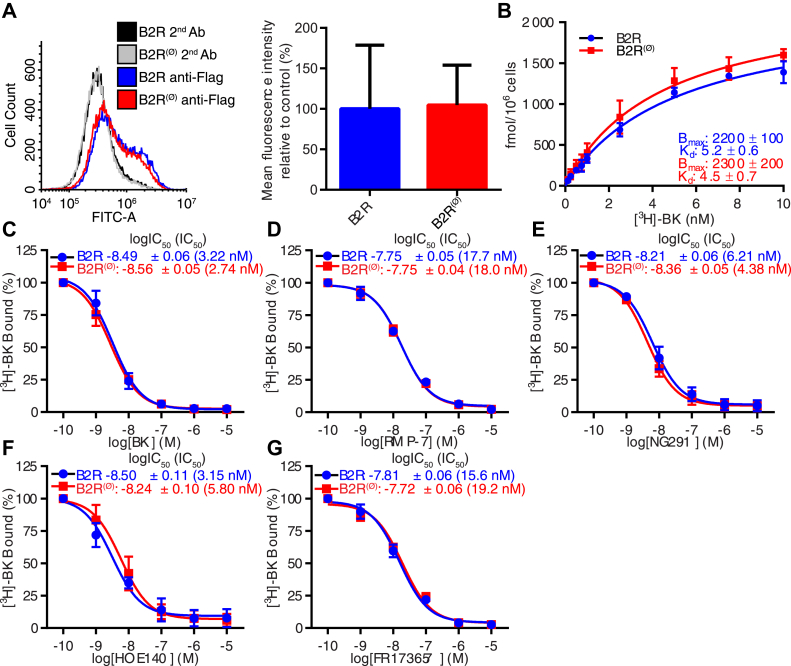
**HEK293A cells transiently transfected with B2R**^**(Ø)**^**mutant (lacking AltB2R) show no difference in B2R cell surface availability and B2R ligand-binding capacity compared to B2R transfected cells.***A*, representative spectrogram (*left*) of ^Flag^B2R and ^Flag^B2R^(Ø)^ detection by FACS. *Black* and *gray*, negative controls with secondary antibodies only; *blue*, ^Flag^B2R transfected cells; *red*, ^Flag^B2R^(Ø)^ transfected cells. Quantification of cell surface B2R expression in HEK293A cells transfected with ^Flag^B2R^(Ø)^ (*red*) compared with control ^Flag^B2R (*blue*) transfected cells (*right*). Data represent mean ± SD, n = 4, unpaired *t*-test, not significant. *B*, cell surface expression of B2R and [^3^H]-BK binding affinity in HEK293A cells transfected with B2R or B2R^(Ø)^. Reported K_d_ are expressed in nM. B_max_ is expressed in fmol/10^6^ cells. Data represent mean ± SD, n = 3, multiple comparison *versus* corresponding ligand concentration using two-way ANOVA with Sidak's correction, unpaired *t*-test for K_d_ and B_max_, not significant. *C*–*G*, competitive displacement of [^3^H]-BK binding by BK (*C*), RMP-7 (*D*), NG291 (*E*), HOE140 (*F*) or FR173657 (*G*) in HEK293A cells transfected with B2R or B2R^(Ø)^. Data represent mean ± SD, n = 3, multiple comparison *versus* corresponding ligand concentration using two-way ANOVA with Sidak's correction, unpaired *t*-test for IC_50_ values, not significant.

Lastly, we sought to determine whether AltB2R could affect cell-surface abundance of B2R and/or could act as a positive allosteric modulator on binding affinities of various selective B2R agonists and antagonists. Fluorescence-activated cell sorter experiments conducted in nonpermeabilized conditions with anti-Flag antibodies on ^Flag^B2R or ^Flag^B2R^(Ø)^ expressing HEK293A cells showed comparable B2R cell-surface expression (Fig. 7*A*). These results were confirmed with saturation experiments depicting nearly identical binding site densities (Bmax values) and radioligand affinities (Kd values of [^3^H]-BK) between B2R- and B2R^(Ø)^-transfected HEK293A cells^(Ø)^ (Fig. 7*B*). Radioligand displacement assays were then performed to determine the influence of AltB2R in agonist/antagonist-binding affinities at B2R. We determined that cells transfected with B2R or B2R^(Ø)^ displayed same affinities to BK (Fig. 7*C*), RMP-7 (Fig. 7*D*), NG291 (Fig. 7*E*), HOE140 (Fig. 7*F*), and FR173657 (Fig. 7*G*).

These results suggest that possible mechanisms underlying potentiation effects of AltB2R may not be ascribed to nonsignaling functions of B2R, such as alternative means to regulate surface abundance of and ligand binding to human B2R. Therefore, main mechanism of AltB2R is likely due to its enhancing effects on selective G-protein-coupling efficiencies of B2R with stimulatory consequences for downstream signaling regulated events including intracellular Ca^2+^ release (*via* IP_3_R) and ERK1/2-MAPK cascades.

### Development and characterization of novel polyclonal antibodies against AltB2R

In order to demonstrate the existence of endogenous AltB2R and allow its detection *in situ*, we generated new polyclonal antibodies against AltB2R. Two antigenic peptides were used to immunize rabbits (Fig. S2*A*). The generated anti-AltB2R antibodies were affinity chromatography-purified and validated for specificity by both western blotting and flow cytometry using mock and AltB2R^Flag^ stable HeLa cell lines (Fig. S2, *B*–*D*). Immunoblot data with the anti-Flag antibody confirmed expression of AltB2R^Flag^ with a strong band appearing at the expected molecular weight (∼17 kDa). This band was not present in mock stable cells (Fig. S2*B*). Notably, the same band was also detected with the new anti-AltB2R antibody in the AltB2R^Flag^ stable cell line, but not in the mock stable cell line. Preadsorption of anti-AltB2R antibodies with antigenic peptides (100× molar excess) prevented the appearance of the band on blotting membranes (data not shown). Moreover, fluorescence-activated cell sorter experiments showed that both anti-Flag (Fig. S2*C*) and anti-AltB2R (Fig. S2*D*) antibodies labeled permeabilized AltB2R^Flag^ expressing cells to a similar extent, with nonspecific binding to mock cells remaining very low. Control experiments using the secondary antibody alone resulted in background fluorescence in both cell lines. Overall, these results demonstrate the ability of our newly developed polyclonal antibodies to specifically recognize both the native and denatured forms of AltB2R.

### Coexpression of AltB2R and B2R in human cancer tissues

**Figure 8:**
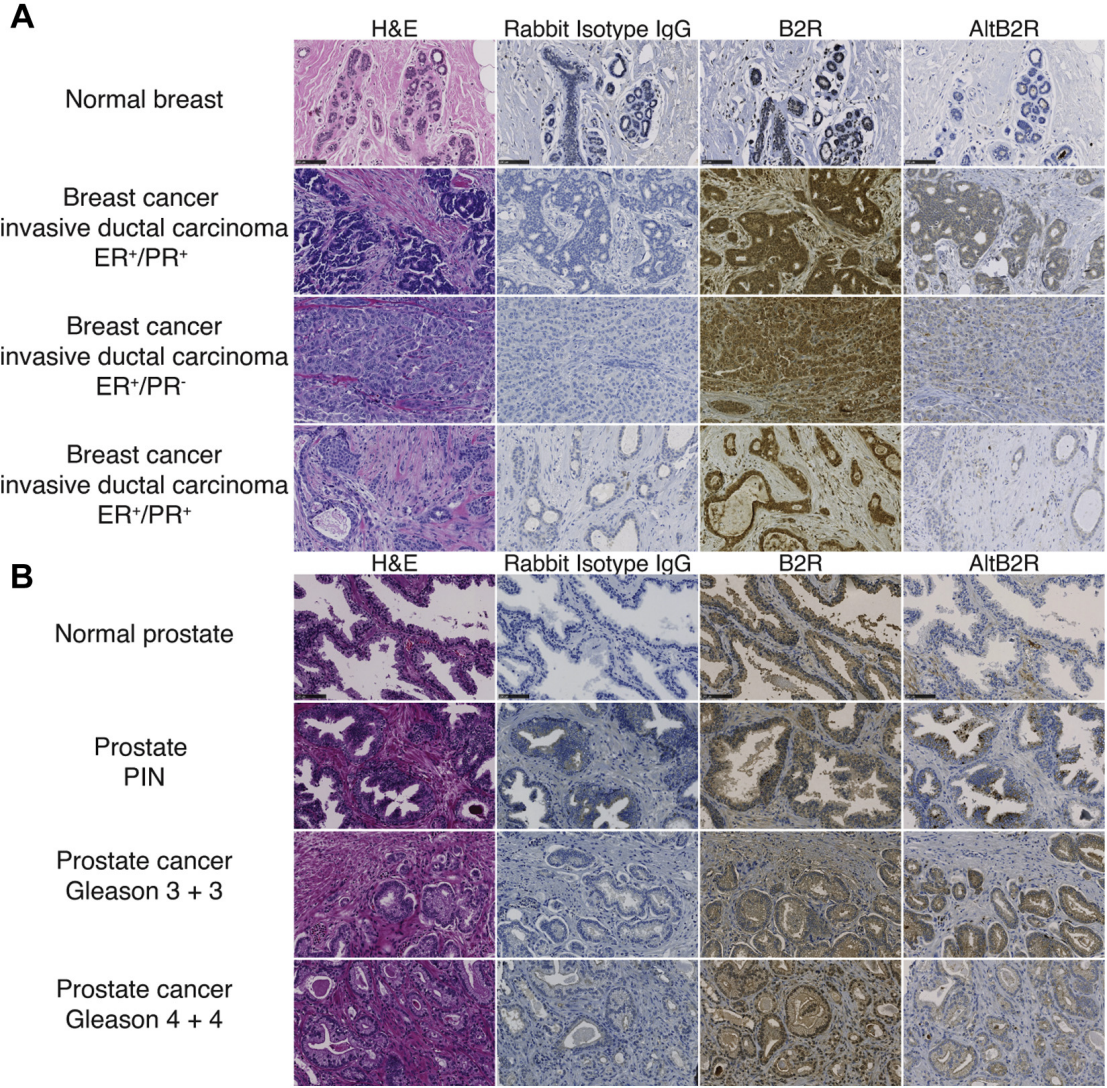
**IHC detection of endogenous AltB2R and B2R in human breast and prostate biopsies.** Representative images of IHC staining of breast (*A*) and prostate (*B*) cancer cases for B2R, AltB2R, and nonimmune IgG. Corresponding H&E images are shown. Advanced stages of breast and prostate cancer tissues showed relatively high levels of B2R and AltB2R immunoreactivities than normal tissues. Scale bar: 100 μm.

Overexpression of kinin B2R has been documented in some types of cancer (20, 21). Immunohistochemistry (IHC) assays were performed to reveal the presence of AltB2R and possible coexpression pattern with B2R on normal and tumoral tissue sections of human breast and prostate using the newly developed anti-AltB2R antibody (Fig. 8). Positive immunoreactivities of both B2R and AltB2R (albeit usually with low intensity for the latter) were found in three invasive ductal carcinoma of breast cancers (Fig. 8*A*). Diffuse perinuclear/cytoplasmic staining was observed in case of B2R while that of AltB2R remained mainly cytoplasmic, indicating a certain degree of colocalization of AltB2R with B2R in breast cancer. By comparison, normal breast tissues showed a much weaker staining for both proteins (Fig. 8*A*). Moreover, both B2R and AltB2R were also detected in prostate cancerous samples with Gleason scores of low (grade 6) to high grade (grade 8) (Fig. 8*B*). Minimal staining of cytoplasmic AltB2R was observed in normal prostate and prostatic intraepithelial neoplasia (PIN), while being somewhat of higher intensity in PIN with staining appearing as punctate (granular/vesicular) and diffuse (Fig. 8*B*). As expected, no or minimal staining was detected with the nonspecific, isotype-matched control antibody in all specimens investigated (Fig. 8, *A* and *B*).

In addition to further describing overexpression of B2R in human solid cancers, our preliminary findings provide initial evidence for the existence and possible relevance of AltB2R in these diseases.

## Discussion

In this study, we discovered a novel hB2R interaction partner protein, AltB2R, encoded by the human *BDKRB2* gene, which acts as a unique positive modulator of hB2R signaling responses. AltB2R is constitutively expressed after transfection of hB2R in host mammalian cells and is endogenously expressed with its reference hB2R in certain human solid cancers, including breast and prostate cancers.

Traditionally, bioinformatic prediction of a “true” ORF was based on the following criteria: the use of an AUG start codon, a minimum length of 100 codons, and a single ORF per transcript (32, 33, 34, 35). However, these criteria substantially underestimate the real protein coding potential of the eukaryotic genome. Indeed, recent large-scale proteogenomic and ribosome profiling studies revealed the irrefutable existence of additional proteins encoded in ORFs smaller than 100 codons present in polycistronic mRNAs and transcripts annotated as noncoding RNAs (36, 37, 38, 39, 40, 41, 42, 43).

Several thousands of alternative proteins have been detected, yet, few of them have known function (44, 45, 46, 47, 48). This is especially true in the case of human GPCR genes (7, 14). This superfamily of membrane proteins is the target of more than 30 to 40% of prescription medicines and remains widely studied for the discovery and validation of new therapeutics against a variety of diseases (49). As indicated in Table S1, we found that the majority of the GPCRs genes (∼81%) in the human genome contain at least one AltORF with a minimum size of 30 codons (4645 AltORFs). Although arbitrary, the threshold of 30 codons makes it possible to limit the size of proteomics databases, a major issue for the proteomics-based discovery of novel proteins (44). It is worth noting that this threshold is not perfect as there are published examples of AltORFs shorter than 30 codons that are translated into bioactive polypeptides (*e.g.*, PEP-7) (14). This suggests an even greater number of possible AltORF present in GPCR genes. Using the prototypical human *BDKRB2* gene as paradigm, we uncovered nine noncanonical ORFs in addition to the currently annotated B2R protein coding sequence (CDS) (Fig. 1). The novel AltORFs within B2R mRNA localize upstream, downstream, or overlapping the B2R CDS in a different reading frame (Fig. 1).

When looking specifically at the purported larger AltORF-resulting protein product (∼17-kDa) derived from the human *BDKRB2* gene, we were able to prove that AltB2R is constitutively coexpressed with hB2R in transfected HeLa cells (Fig. 1). Hence, both proteins are produced from the B2R CDS. Moreover, our IF, WB, and IHC results confirmed the cytosolic distribution of AltB2R, in cultured human cells and in human cancer tissue biopsies (Figs. 2, 3 and 8). AltB2R showed also good colocalization with hB2R, within multiple punctate speckles, and apparent physical interaction between both proteins at the plasma membrane and within intracellular perinuclear compartments. The latter are presumed to be ER/Golgi complexes and common endosomes, as demonstrated by IF, co-IP, and BiFC experiments (Figs. 2 and 3). Since visualization of spatiotemporal dynamics of AltB2R-B2R interactions has not been carried out within living cells, it remains to be seen whether these interactions are transient or permanent in relation to the cellular compartments.

The current study was conducted with human cells transfected with expression vectors encoding AltB2R alone (HeLa) and hB2R with or without coexpression of AltB2R (HEK293A). This allowed us to interrogate for the first time B2R mediating signaling events that occur in the presence or absence of its newly identified potential interacting partner, AltB2R. Our findings provided early confirmation of functional coupling of agonist-stimulated hB2R to the G proteins Gα_q_, Gα_i2/3_ as well as to its ability to recruit β_2_-arrestins and activate the classical IP_3_, Ca^2+^, and ERK1/2 messenger signaling cascades (Figure 4, Figure 5, Figure 6) (20, 22, 26, 50, 51). Importantly, they also highlighted a distinct property of AltB2R in imparting significant positive modulatory effects on agonist-dependent B2R activity leading to higher signal amplitudes of MAPK activation. Notably, this outcome is the opposite of that reported for rat AT_1a_R encoded-PEP7, which exerts inhibitory actions on β-arrestin-dependent MAPK activation (14). Looking at the most important upstream components contributing to MAPK activation, up to proximal activator of this pathway, G proteins, we found that AltB2R caused signal enhancement in all of these (Figure 4, Figure 5, Figure 6). Specifically, the maximum G protein-responses to agonist stimulation (efficacy) were increased, whereas agonist potencies (reflecting apparent affinity estimates) remained unchanged (Fig. 6). This is congruent with the similar binding affinity results observed for the pool of relevant B2R agonists/antagonists tested between HEK293A cells expressing wild-type B2R and mutant B2R^(Ø)^ (Fig. 7, *C*–*G*). Other possible factors that may account for the observed phenomenon on MAPK activity, such as changes in basal cell surface B2R density or in efficiencies of cognate agonists at promoting B2R internalization and β-arrestin 2 recruitment, were ruled out by our experiments (Figs. 6, *D*–*F* and 7, *A* and *B*). Altogether, these results point to a pivotal role of AltB2R in modifying levels of activated B2R-G protein (G_q_, G_i_) interactions, facilitating heterotrimeric G protein dissociation into Gα-GTP and Gβγ subunits and subsequent signaling pathways involved in activation of MAPK ERK1/2. For this to happen, AltB2R must be in proximity with or even be precoupled to hB2R.

Interestingly, AltB2R effects appear to be specific for hB2R since it had no influence on hB1R-mediated MAPK ERK1/2 activation (Fig. S4); this is in accordance with the lack of interaction observed between human B1R and AltB2R proteins (Fig. S3*A*). A further investigation using mutagenesis experiments with chimeric B1R containing fragments of intracellular loops and/or C-terminal domains of hB2R may shed light on the way AltB2R and B2R functionally interact. Obviously, a large number of the currently described pathways leading to MAPK stimulation by B2R have not been considered in the present study (*e.g.*, G_0_/G_12_/G_13_ proteins, disassociated G protein βγ-subunits, epidermal growth factor receptor (EGFR) transactivation, heterodimerization) (20, 21, 22). Further investigation is required to elucidate the function of AltB2R on other G protein-dependent or independent-B2R signaling pathways linked to distinct phenotypic outcomes.

Finally, another major key finding of this study was the IHC data showing endogenous expression of AltB2R and co-overexpression of B2R and AltB2R in some cancer types. The coexpression and partial colocalization of AltB2R and B2R leave open the possibility for exploring complex parallel but intertwined relationship between these two proteins in cancers. Such work is ongoing in our laboratories and will be the subject of another study. The associations of AltB2R and B2R can be considered biologically plausible based on our collected data obtained from *in vitro* cellular models.

Our findings raise intriguing fundamental questions about the molecular mechanisms underlying the expression and functions of relevant AltB2R in physiological and pathological states as well as to the possibility of manipulating them for therapeutic applications, such as a means to reduce or improve sensitivity of cellular responses to endogenous specific GPCR agonists. Undoubtedly, the study of AltORFs can offer a lot of interesting basic science and clinical perspectives, with tremendous implications on GPCR research, given that deregulation of gene expression profiles and numbers of polymorphisms of GPCRs have emerged as an important factor in many pathologies. For hB2R, these include, for instance, cancer, hypertension, ischemic heart disease, insulin resistance, ACEI-induced cough, and osteoarthritis (18). Special attention should equally be paid to cell and animal experimentations involving gene expression studies (*e.g.*, cDNA transfection, knock-down, knock-out, transgenes, and gene therapy) since they could result in the expression, downregulation or deletion of the reference GPCR protein and unnoticed alternative proteins, leading to confounding results (6).

In summary, here we identified AltB2R as a new member of the AltORF family of human GPCR genes and elucidated potential molecular mechanistic basis of AltB2R in amplifying agonist-dependent B2R signaling pathways. If substantiated, the new concept of alternate proteome for B2R, which could be generalizable to other GPCRs, may set forth new vistas, challenges, and opportunities in GPCR biology, diseases, and drug discovery.

## Experimental procedures

### Plasmid construction and cloning

All primer sequences are outlined in Table S3. The PCR products were inserted into the BamHI restriction site of pcDNA3.1- using the Gibson assembly kit (New England BioLabs, E2611 L) according to the manufacturer's instructions. The B2R^Flag^-VN, B1R^Flag^-VC, VN-AltB2R^HA^, and VC-AltB2R^HA^ PCR products were inserted into the BamHI restriction site of pVN-C, pVC-C, pVN-N, and pVC-N, respectively. VN-C, VC-C, VN-N, and VC-N fragments were amplified by PCR (from templates provided by Pr. Jean-Bernard Denault (Université de Sherbrooke)) and inserted into the BamHI restriction site of pcDNA3.1- to create pVN-C, pVC-C, pVN-N, and pVC-N. BRET biosensor constructs (Gαq, Gαi2, Gαi3, β-arrestin 2) were provided by Pr. Richard Leduc (Université de Sherbrooke). The B2R^(Ø)^ construct, corresponding to the mutant human B2R lacking the concomitant expression of AltB2R, was obtained by using the Q5 Site-Directed Mutagenesis Kit (New England BioLabs, MS0554S) on B2R. gBlock gene fragment of AltB2R^-3Flag^ was inserted into the BamHI restriction site of pcDNA3.1- using the Gibson assembly kit. AltB2R^Flag^ PCR product was inserted into the BamHI restriction site of pLenti6 V5a, which was provided by Pr. Fernand-Pierre Gendron (Université de Sherbrooke). Primers and sequence-verified gBlock gene fragments were purchased from IDT. All constructs were sequenced in both orientations.

### Chemical reagents and antibodies

Primary antibodies used were monoclonal anti-γ-Tubulin (Sigma, clone GTU-88, mouse), anti-Flag (Sigma, clone M2, mouse), anti-HA (Invitrogen, clone #2-2.2.14, mouse), anti-CD71 (Invitrogen, clone H68.4, mouse), anti-GAPDH (Santa Cruz Biothechnology, clone #0411, mouse) and polyclonal anti-HA (Immune Biosolutions, Y00001-002, chicken), anti-HA (Abcam, ab9110, rabbit), anti-Lamin A/C (Santa Cruz Biotechnology, N-18, goat), anti-human B2R LS-A797 (LifeSpan Biosciences, rabbit), anti-NPTII (Millipore Sigma, #06-747, rabbit), anti-pERK1/2 (Cell Signaling, #9101, rabbit), anti-ERK (Santa Cruz Biotechnology, C-16, rabbit), and negative control rabbit Ig fraction (Dako). Secondary antibodies used were anti-mouse IgG-HRP (Cell Signaling, #7076, horse), anti-rabbit IgG-HRP (Cell Signaling, #7074, goat), anti-goat IgG-HRP (Abcam, #6885, donkey), Veriblot-HRP (Abcam, ab131366), anti-rabbit Alexa Fluor405 (ThermoFisher Scientific, #A-31553, goat), anti-mouse Alexa Fluor488 (Invitrogen, A-11017, goat), anti-rabbit Alexa Fluor488 (Invitrogen, A-11008, goat), anti-chicken Atto594 (Immune Biosolutions, Y01038-594, goat), anti-chicken-Atto390 (Immune Biosolutions, Y01038-390, goat), anti-mouse-Alexa Fluor647 (Cell Signaling, #4410, goat). A custom antibody was raised against two synthetic peptides from AltB2R (H-RDLPGEPGRSRPDPGL-OH, H-DAGEHRPLPGPGEN-OH) in a rabbit and purified by antigen peptide affinity chromatography by Abcam. The rabbit anti-human B2R antiserum AS277-83 (targeting eight distinct epitopes on the extra- and intracellular domains of B2R) was kindly provided by Pr. Werner Muller-Esterl (University Frankfurt). Target specificity of the two anti-B2R antibodies LS-A797 and AS277-83 has been reported elsewhere (52, 53). Quality control experiments using dynamic light scattering (DLS) analyses confirmed structural integrities of anti-AltB2R and -B2R antibody sources (data not shown). All other reagents were obtained from Sigma-Aldrich, unless otherwise stated.

### Solid-phase peptide synthesis

The peptides bradykinin [BK; H-Arg^1^-Pro^2^-Pro^3^-Gly^4^-Phe^5^-Ser^6^-Pro^7^-Phe^8^-Arg^9^-OH)], NG291 ([Hyp^3^,Thi^5^,^N^Chg^7^,Thi^8^]-BK; B2R agonist), HOE140 (dArg[Hyp^3^,Thi^5^,dtic^7^,Oic^8^]-BK; B2R antagonist), and NG29 (SarLys[dphe^8^]desArg^9^-BK; B1R agonist) were assembled on a solid support by an automated Pioneer peptide synthesizer using 9-fluorenylmethyoxy-carbonyl chemistry (54, 55). Peptides were purified by analytical reversed-phase high-performance liquid chromatography (RP-HPLC) and were >95% pure, with expected mass spectra. The peptide B2R agonist RMP-7 ([Hyp^3^,Thi^5^,(4-Me)Tyr^8^(ΨCH_2_NH)Arg^9^]-BK) was supplied by Bachem Bioscience. Peptides were stored in the powder form at −20 °C. Stock solutions (10 mM) of peptides were also prepared in Nanopure water. The nonpeptide B2R antagonist FR173657 was kindly provided by Astellas Pharma Inc (formerly Fujisawa Pharmaceutical Co) and was prepared in 50% dimethyl sulfoxide (DMSO)/50% Nanopure water.

### Cell culture and transfections

HeLa, HEK293T, and HEK293A cells were maintained in Dulbecco's modified Eagle's medium (DMEM) containing 10% fetal bovine serum (FBS) (Wisent, #080-450) and 1% antibiotic-antimycotic (Wisent, #450-115-EL) under standard conditions. HEK293A cells were transiently transfected with the pcDNA3.1-vector containing the cDNA of human B2R or B2R^(Ø)^, using polyethylenimine (PEI) or JetPrime (Polyplus). HeLa cells were transiently transfected with B2R and AltB2R constructs using GeneCellin (Bulldog Bio Inc) or JetPrime (Polyplus) transfection reagent, as per the manufacturer’s instructions, and incubated 24 h at 37 °C, unless otherwise stated.

### Western blot

HeLa cells from 6-well plates were washed with phosphate-buffered saline (PBS), scraped with 150 μl Laemmli buffer containing 2-Mercaptoethanol (βME), sonicated, and heated at 56 °C for 10 min. Proteins were separated in a 12% or 15% SDS-PAGE. After transfer, PVDF membranes were probed with anti-Tubulin (1/10,000), anti-NPTII (1/4000), anti-Flag (1/1000), and anti-HA (1/5000, mouse) antibodies overnight at 4 °C. After three washings, membranes were incubated for 1 h at room temperature with goat anti-rabbit IgG-HRP (1/10,000) or anti-mouse IgG-HRP (1/10,000) antibodies. Proteins were detected with the Western Lightning ECL reagent (Perkin Elmer, NEL103001EA) according to the manufacturer’s instructions and the ImageQuant LAS 4000 system (GE Healthcare, # 28955811).

### Cell fractionation and organelle isolation

Cell fractionation on HeLa cells was carried out using Cell Signaling Technology's Cell Fractionation Kit (#9038) according to the manufacturer's instructions. Protein fractions were then separated in a 12% or 15% SDS-PAGE. Membranes were probed with anti-CD71 (1/2000), anti-Lamin A/C (1/1000), anti-GAPDH (1/10,000), and anti-HA (1/5000, mouse) antibodies overnight at 4 °C. Proteins were detected as described above with anti-goat IgG-HRP (1/5000) antibodies.

### Immunofluorescence and confocal microscopy

Immunofluorescence and confocal analyses were carried out as previously described with slight modifications (3). Briefly, HeLa cells were fixed for 20 min with 4% paraformaldehyde (PFA), then incubated for 30 min in 10% normal goat serum (Wisent, #053-150) and 0.1% saponin (Sigma) in PBS. Cells were incubated in the same buffer with anti-Flag (1/1000) and anti-HA (1/500, chicken) primary antibodies and with anti-mouse-Alexa Fluor488 (1/1000) and anti-chicken-Atto594 (1/500) or anti-mouse-Alexa Fluor647 (1/1000) and anti-chicken-Atto390 (1/100) secondary antibodies. Cells were then examined with a scanning confocal microscope (FV1000; Olympus) coupled to an inverted microscope with a 63x oil-immersion objective (Olympus).

For live-cell staining, HeLa cells grown on coverslips were washed with DMEM and incubated for 30 min on ice with anti-Flag (1/1000) and anti-HA (1/500, chicken) in DMEM. After washing, cells were incubated for 30 min on ice with anti-mouse-Alexa Fluor488 (1/1000) and anti-chicken-Atto594 (1/500) antibodies diluted in DMEM. After washing, cells were processed as described above.

### Generation and validation of stable cell lines

HEK293T cells were cotransfected with pCMV R8.2 (addgene, #12263), pMD2.G (addgene, #12259) and pLenti6V5A-AltB2RFlag (or the empty vector for Mock cell line) using Lipofectamine 2000 as per the manufacturer’s instructions. After 48 h at 37 °C, the cell media was filtered through 0.45 μM pores and the viral supernatant collected. Polybrene (1 μg/ml) and the viral supernatant (2 ml) were added to HeLa cells grown in 6-well plates, and the cells were incubated at 37 °C for 48 h. The media was then replaced with media containing 2 μg/ml of blasticidin (Wisent, #450-190-XL), and the cells were incubated at 37 °C for another 24 h. Cells were then passaged while gradually increasing the blasticidin concentration at every passage (2, 4, 8, 15 μg/ml). Stable cell lines were maintained in DMEM containing 10% FBS, 1% antimicrobial/antimycoplasma and 15 μg/ml of blasticidin. Stable cells from a 6-well plate were washed with PBS, scraped with RIPA (50 mM Tris-HCl, pH 7.5, 0.1% SDS, 1% Sodium Deoxycholate, 1% Triton X-100, complete protease inhibitors (Roche, #11873580001)), and sonicated. Samples were quantified using BCA protein assay reagent and 100 μg of protein was diluted in Laemmli (βME). Samples were boiled for 5 min at 95 °C and processed for western blot analysis as described above with anti-AltB2R (1/1000; 1:1 stock solution).

### Bimolecular fluorescence complementation (BiFC) assays

HeLa cells cotransfected with B2R^Flag^-VN and VC-AltB2R^HA^ were grown on coverslips for 24 h in DMEM plus 10% FBS. For immunostaining of hB2R and AltB2R, cells were fixed with 4% PFA, permeabilized with 0.15% Triton X-100 in PBS, immunostained with anti-Flag (1/1000) and anti-HA (1/500, chicken) or anti-HA (1/1000, rabbit) primary antibodies, and then with fluorescently labeled secondary antibodies, anti-mouse- Alexa Fluor647 (1/1000) and anti-chicken- Atto390 (1/100). All coverslips were mounted onto glass slides using SlowFade in glycerol/PBS. Fluorescence signals of Venus (gray) or antibodies were observed with a laser scanning confocal microscope, as described above.

### Flow cytometry

HEK293A cells transiently transfected with ^Flag^B2R or ^Flag^B2R^(Ø)^ were washed with PBS, harvested with Accutase (Gibco, A1110501) centrifuged 5 min at 800 rpm, resuspended in PBS, counted and 300,000 cells per condition were transferred to a new tube. Cells were centrifuged for 5 min at 800 rpm, resuspended in 500 μl PBS, and fixed for 20 min after adding 500 μl of 4% PFA. Cells were centrifuged for 5 min at 800 rpm at 4 °C, washed with 0.1 M glycine in PBS, centrifuged for 5 min at 800 rpm at 4 °C, resuspended in Cyto buffer (1% bovine serum albumin (BSA; Wisent, #800-095-EG) and 0.1% saponin in PBS), and incubated for 20 min on ice. Cells were split into individual tubes (300,000 cells per condition) and were incubated in the presence or absence of the primary antibody, anti-Flag (1/1000) or anti-AltB2R (1/100,000), overnight at 4 °C with rocking). After two wash-quick spin steps with 1 ml Cyto buffer, cells were incubated for 1 h at 4 °C in the dark with either anti-mouse-488 (1/10,000) or anti-rabbit-488 (1/10,000) antibody. Cells were processed through two wash-quick spin steps with 1 ml Cyto buffer, resuspended in 300 μl PBS, and analyzed by a CytoFLEX flow cytometer (Beckman Coulter). A minimum of 30,000 gated events by sample were acquired. Fluorescence intensity distribution was analyzed with the CytExpert software (Beckman Coulter). A mean fluorescence intensity (MFI) recorded for each cell population was subtracted by the respective background fluorescence observed in the control (secondary antibody alone). Results are expressed as MFI relative to control cells (%).

### Receptor binding assays

Radioligand competition binding assay was performed using adherent living HEK293A cells transiently transfected with B2R or B2R^(Ø)^, as we previously described (54). Displacement binding was carried out by incubating the cells with the competing B2R agonists BK, RMP-7, and NG291 or the B2R antagonists HOE140 and FR173657 (in the range of 0.01 nM–10,000 nM) and the radioligand [^3^H]-BK (2 nM) for 2 h at 4 °C. Saturation binding assays were performed using adherent living HEK293A cells transiently transfected with B2R or B2R^(Ø)^. Cells were washed twice with binding buffer (DMEM supplement with 0.1% BSA, 10 μM captopril, 5 μM thiorphan, 20 μM mergetpa and 0.1% NaN_3_) and incubated with [^3^H]-BK (in the range of 0.125 nM–10 nM) for 2 h at 4 °C. Nonspecific binding was determined in the presence of 10 μM HOE140. After the incubation time, cells were washed twice with ice-cold PBS, lysed with 200 μl of 0.1 N sodium hydroxide, and transferred into scintillation vials containing 4 ml scintillation liquid. The radioactivity was measured with a β-counter and data were analyzed using GraphPad 6.01 (GraphPad Software, Inc).

### ELISA assays

Receptor internalization kinetics were determined by cell surface ELISA on transiently transfected HEK293A cells with ^Flag^B2R or ^Flag^B2R^(Ø)^. Cells were grown in 24-well plates for 48 h after transient transfection. Cells were washed twice with HBSS, then stimulated with 1 μM RMP-7 or 1 μM NG291 in HBSS (in the time range of 0 min–90 min). The reaction was terminated by incubating the cells for 5 min with 2% PFA at room temperature. Following two washes in TBS (50 mM Tris-HCl pH 7.5, 150 mM NaCl), cells were incubated for 30 min at room temperature in 1% BSA in TBS, followed by 30 min at room temperature with the anti-Flag (1/1000) in 1% BSA in TBS. Cells were washed twice with TBS and incubated with 250 μl 3,3',5,5'-tetramethylbenzidine (TMB; Sigma #T2885) for 15 min at room temperature. The reaction was stopped with 250 μl HCl 2 N. After transferring 200 μl to a microplate, the plate was read at 450 nm. Control wells transfected with an empty vector and stimulated for 90 min with the agonist were used to subtract the background noise and data were normalized with the untreated cells.

### Bioluminescence resonance energy transfer (BRET) assays

BRET assays were performed as previously described (56). Briefly, HEK293A cells were grown in 96-well plates for 48 h after transient transfection with 10 μg BRET constructs (Gα_q_-RLuc2, Gβ_1_, and GFP10-Gγ_1_) (Gα_i2_-Rluc2, Gβ_1_, and GFP10-Gγ_1_) (Gα_i3_-Rluc2, Gβ_1_, and GFP10-Gγ_1_) (β-arrestin2-Rluc2 and rGFP-CAAX) and B2R or B2R^(Ø)^. Cells were washed twice with stimulation buffer (10 mM HEPES pH 7.4, 1 mM CaCl_2_, 0.5 mM MgCl_2_, 4.2 mM KCl, 146 mM NaCl, 5.5 mM glucose) and incubated for 1 h in 80 μl stimulation buffer at room temperature. Cells were incubated for 10 min with 10 μl coelenterazine (5 μM) (Sigma, #C2230) before being stimulated with 10 μl NG291 (concentrations in the range of 1 pM–10 μM) for 10 min. BRET readings were collected in the 400 to 450 nM (Rluc2) and 500 to 550 nM (GFP10) windows, and BRET signal was calculated as a ratio of GFP10 on Rluc2. Data were normalized with unstimulated cells.

### Intracellular calcium mobilization assays

Stable HeLa cells expressing AltB2R were grown on 25 mm coverslips 24 h prior to the experiment. Cells were washed three times with Tyrode buffer (5 mM HEPES, 136 mM NaCl, 2.7 mM KCl, 1 mM MgCl2, 1.9 mM CaCl2, 5.6 mM glucose, pH 7.4 adjusted with Tris base, 310 mOsm) supplemented with 0.1% BSA (Sigma, St Louis) and 2.5 mM probenecid and then incubated with the calcium probe, Fluo-4/AM (Molecular Probes) at a final concentration of 13 μM at room temperature for 1 h. After the incubation period, the cells were washed three times in the Tyrode’s buffer containing 2.5 mM probenecid (to retain the calcium indicator dye within cells). Cells were visualized with a Bio-Rad confocal krypton–argon and ultraviolet laser system as previously described (57, 58). All confocal microscope settings (laser line intensity, photometric gain, photomultiplier tube (PMT) settings, and filter attenuation) were kept rigorously constant throughout all the experiments. Quantitative 3D confocal microscopy was used to monitor intracellular free calcium. The basal fluorescence intensity for intracellular [Ca2+] levels of unstimulated cells was monitored until steady-state level was achieved, then the B2R agonist RMP-7 (1 μM) was applied to the cells, and variations of intracellular [Ca2+] were monitored. At the end of each experiment, the nucleus was stained with 100 nM of live cell nucleic acid stain Syto-11 (Molecular Probes). Scanned images were transferred onto a Silicon Graphics workstation equipped with Molecular Dynamics’ ImageSpace analysis and Volume Workbench software modules, which allow the quantification of intracellular free Ca2+ per μm^3^.

### MAPK (ERK1/2) phosphorylation assays

Stable HeLa cell lines or HEK293A cells transiently transfected with B2R or B2R^(Ø)^ were grown in 6-well plates and stimulated with 1 μM RMP-7. After treatment, cells were washed with ice-cold PBS and scraped in PBS. Cells were centrifuged for 5 min at 10,000*g* at 4 °C, lysed in RIPA buffer containing PhosStop (Roche, #04906845001), and samples were quantified using BCA protein assay reagent. For each sample, 40 μg of protein was solubilized in Laemmli buffer with βME and separated in a 12% SDS-PAGE. Western blots were carried out as described above with anti-pERK1/2 (1/2000) antibodies. Membranes were then stripped by boiling for 2 min in the microwave in 50 ml deionized water and probed with anti-total ERK1/2 (1/50,000) antibodies. Densitometric analysis was conducted using ImagePro Plus 5.1 (Media Cybernetic). The results are presented as intensities of phospho-ERK1/2 bands relative to total ERK1/2 bands.

ERK1/2 phosphorylation was also monitored in HEK293A cells using the Perkin Elmer’s AlphaLISA SureFire Ultra phospho-ERK1/2 (Thr202/Tyr204) assay kit. Cells were seeded in 10 mm petri dish (at 60–80% confluency) and transfected with 5 μg B2R or B2R^(Ø)^ plasmids using JetPrime reagent (Polyplus) for 24 h at 37 °C. Cells were subsequently harvested, plated into 96-well plate (80–100 × 10^3^ cells/well), and cultured for an additional 24 h in complete DMEM. Thereafter, cells were starved overnight in phenol-free DMEM and then stimulated with 1 μM RMP-7 or NG291 at various times. Stimulation was ended by the addition of 20 μl of 5× lysis buffer. Lysates were incubated at room temperature for 10 min on a plate shaker and frozen overnight at −20 °C. A total of 5 μl of the lysate was used for analysis. The readings were performed on a Mithras LB 943 multimode microplate reader from Berthold Technologies.

### Inositol phosphate production assays

HEK293A cells transiently transfected with 10 μg B2R or B2R^(Ø)^plasmids for 48 h were washed with PBS at room temperature, harvested with Accutase, and distributed at 15,000 cells/well in a white 384-well plate in stimulation buffer at 37 °C for 30 min with increasing concentrations of B2R agonists, NG291 and RMP-7. Inositol monophosphate (IP_1_) production was determined using the IP-One Gq assay kits (Cisbio, #62IPAPEB), according to the manufacturer's instructions. Fluorescence resonance energy transfer (FRET) signals were measured with a Tecan M1000 plate reader.

### Human tissue samples and immunohistochemical (IHC) assays

Breast and prostate cancer tissue and normal adjacent tissue (except for normal breast tissues, which were taken from reduction mammaplasty) were obtained from patients who underwent surgical resection of their primary tumor at the Hôtel-Dieu Hospital (Québec, Canada) or the Centre Hospitalier Universitaire de Sherbrooke (CHUS). All tumor specimens were collected from treatment-naïve patients. Patients agreed to participate and freely signed a consent form. The study was conducted in accordance with the Declaration of Helsinki, and the protocol was approved by the Ethics committee of the Centre de Recherche du CHUS (Project #1997-7, 97-14). The tumors and normal tissues were fixed in 10% phosphate-buffered formalin for 24 h, embedded in paraffin, cut into 4 to 5-μm thick sections, and mounted on positively charged slides. For histological confirmation, the sections were stained with haematoxylin and eosin.

IHC staining was performed with an automated system (Dako Autostainer plus) using the Envision Flex High pH visualization system (Dako) (59). Sections were stained with the anti-AltB2R (1/100), anti-B2R AS277-83 (1/800) or the negative control consisting of a nonimmunized rabbit immunoglobulin fraction (Dako) (1/100). The EnVision FLEX/HRP (Dako) reagent containing a dextran polymer conjugated to anti-mouse Ig and anti-rabbit Ig and HRP was used as the secondary antibody. Diaminobenzidine (DAB) (Roche) was used as chromogen. Nuclei were counterstained with hematoxylin. Slides were scanned using a Hamamatsu Nanozoomer 2.0-RS whole slide imager at 40x magnification. Pictures were then analyzed with the Hamamatsu NDP view 2 software.

### Statistical analysis

Data are presented as mean ± standard deviation (SD). Data were compared using unpaired two-tailed Student’s *t*-tests, one/two-way ANOVA with Sidak's correction or with Dunnett's tests, where appropriate. Statistical significance was set at *p* <0.05. Statistical analysis was performed using GraphPad Prism 6.01 (GraphPad Software, Inc).

## Data availability

Any data that support the findings of this study are included within the article and its supplementary information files.

## Conflict of interest

The authors declare that they have no conflicts of interest with the contents of this article
