# Supplementary material for: Potentiation of B2 receptor signaling by AltB2R, a newly identified alternative protein encoded in the human bradykinin B2 receptor gene
Source: J Biol Chem. 2021 Jan 23;296:100329. doi: 10.1016/j.jbc.2021.100329 (PMC7949122; doi:10.1016/j.jbc.2021.100329)
Supplement: Supplemental Figures S1–S4 [file mmc4.pdf]

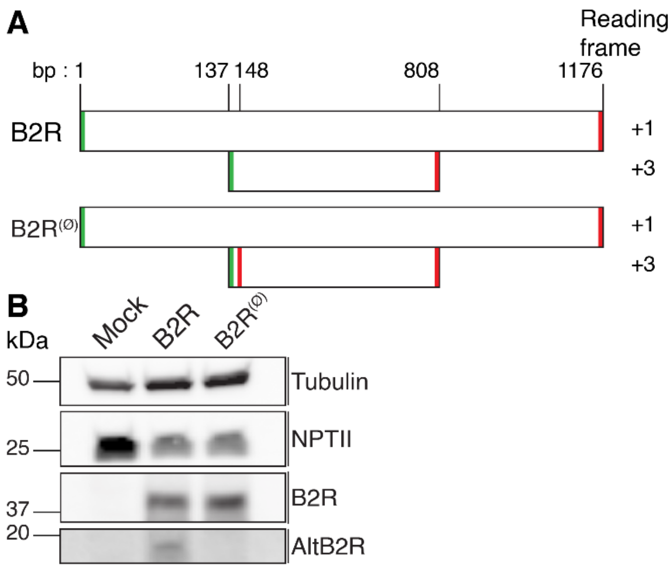

Figure S1. HEK293A cells transfected with B2R$^{(Ø)}$ mutant show no expression of AltB2R compared to B2R transfected cells. **A)** Schematic representation of the strategy to construct plasmids encoding the silent mutation of B2R in B2R$^{(Ø)}$, unaffecting expression of B2R while preventing the expression of AltB2R. **B)** Representative western blot images providing confirmation of the presence and absence of AltB2R in HEK293A cells expressing wild-type B2R and mutant B2R$^{(Ø)}$, respectively. Tubulin serves as a loading control and NPTII serves as a transfection control. Membranes were probed with primary antibodies at the following dilutions: anti-tubulin (1/ 10 000), anti-NPTII (1/ 4 000), anti-B2R LS-A797 (1/1 000) and anti-AltB2R (1/1 000) antibodies. Antibody used for the detection of AltB2R is fully described in following Fig. S2. Proteins were detected using either anti-rabbit or anti-mouse IgG-HRP (1/10 000) antibodies, as described under Experimental Procedures.

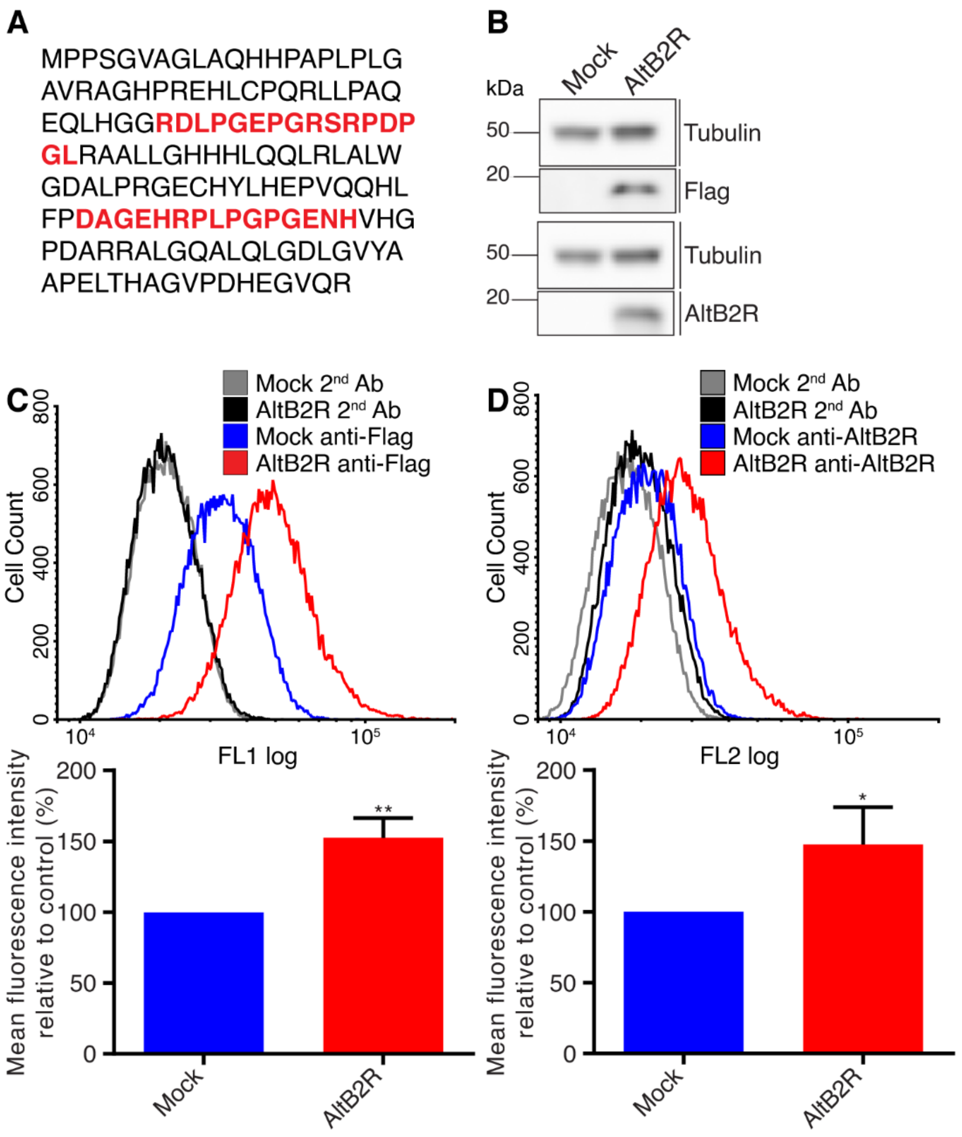

Figure S2. A custom antibody raised against two peptides of AltB2R detects both native and denatured states of AltB2R in stable HeLa cell lines. **A)** Amino acid sequence of AltB2R. The bold, red text represents the two antigen sequences chosen to produce the custom antibody. **B)** Western blot analysis of AltB2R^{Flag} stable cell line using anti-Flag or anti-AltB2R antibodies. **C, D)** Representative FACS histograms showing Flag (**C**, top) or AltB2R (**D**, top) detection under permeabilized conditions. Black and grey: negative controls with secondary antibodies only; blue: Mock cell line; red: AltB2R^{Flag} cell line. Quantification of Flag (**C**, bottom) or AltB2R (**D**, bottom) relative mean fluorescence intensity. Data represent mean ± SD, unpaired t-test, n=4, * $p<0.05$, ** $p<0.01$.

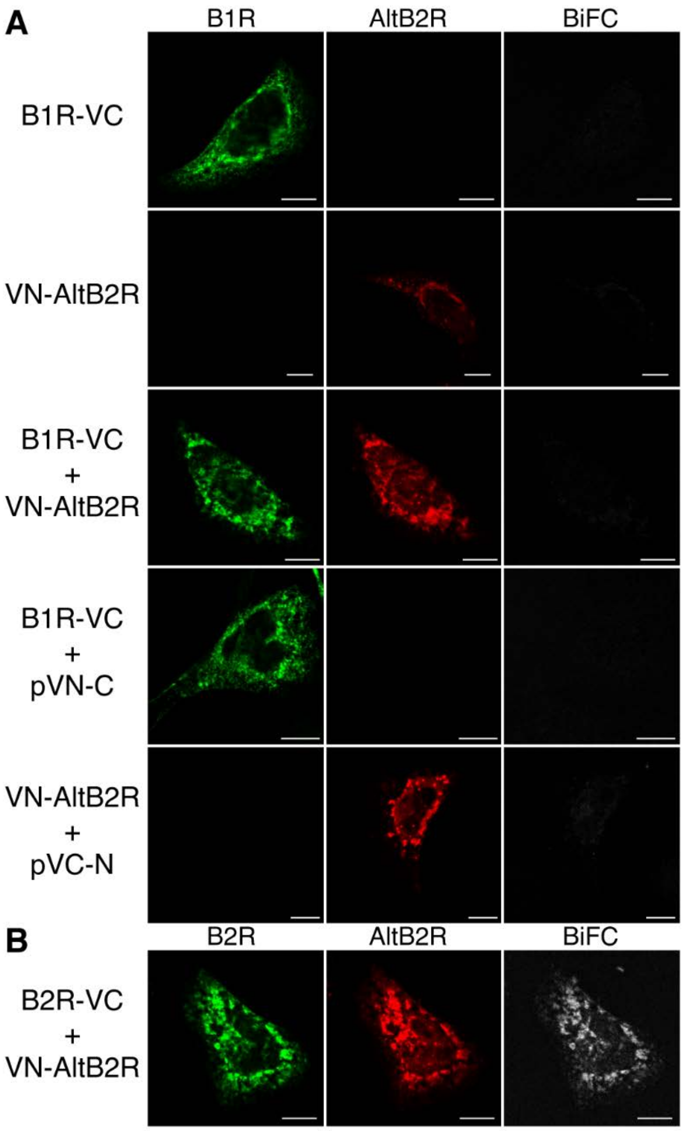

Figure S3. Lack of interaction between AltB2R and human B1R in HeLa cells, as determined by BiFC assays. **A)** Expression of $B1R^{Flag}$-VC and VN-$AltB2R^{HA}$ results in no detectable BiFC signal in transiently transfected HeLa cells. No detectable BiFC signal from non-specific assembly of the Venus protein with tagged B1R or AltB2R. Immunocytological localization of B1R and AltB2R were determined using primary antibodies anti-Flag and anti-HA (rabbit) and secondary anti-mouse-Alexa Fluor®647 and anti-rabbit-Alexa Fluor®405. **B)** Confirmation experiments (within the same study) showing positive interactions between $B2R^{Flag}$-VC and VN-$AltB2R^{HA}$ in transfected HeLa cells using BiFC assays. Scale bar: 10 µm. (A-B) Representative single confocal mid-section images are shown.

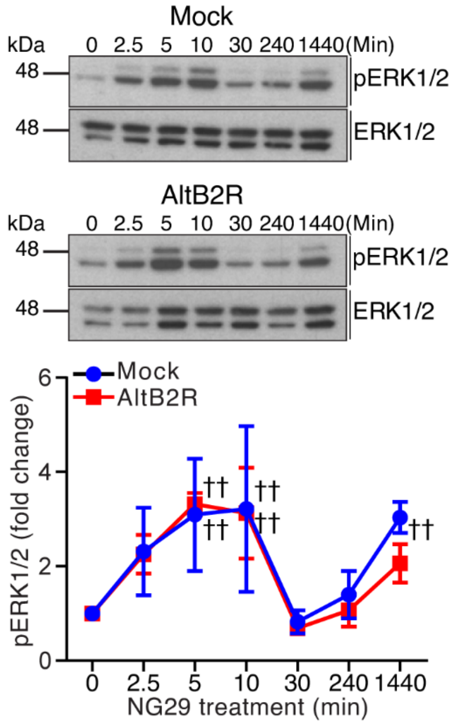

Figure S4. Stable AltB2R^{Flag} expressing HeLa cells exhibit a similar pattern of MAPK ERK1/2 activation compared to stable Mock cells following stimulation of endogenous B1R by 1 µM NG29. Representative Western Blot images (top) of phosphorylated ERK1/2 and total ERK1/2 at different stimulation times. Activation kinetics (bottom) of ERK1/2 following B1R stimulation by 1 µM NG29 in Mock (blue) and AltB2R^{Flag} (red) stable cell lines. Data represent mean ± SD, n=3, multiple comparison versus control (0 min) using two-way ANOVA with Dunnett's correction, †† $p<0.01$, multiple comparison versus corresponding time point using two-way ANOVA with Sidak's correction, not significant.
